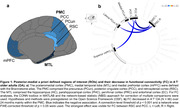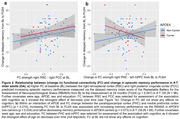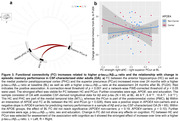# Longitudinal posterior‐medial functional connectivity changes with age and Alzheimer’s pathology are differentially related to memory performance depending on APOE genotype

**DOI:** 10.1002/alz.093962

**Published:** 2025-01-09

**Authors:** Larissa Fischer, Eóin N. Molloy, Jenna N. Adams, Jennifer Tremblay‐Mercier, Jordana Remz, Alexa Pichet Binette, Sylvia Villeneuve, Anne Maass

**Affiliations:** ^1^ German Center for Neurodegenerative Diseases (DZNE), Magdeburg Germany; ^2^ University of California, Irvine, Irvine, CA USA; ^3^ Douglas Mental Health University Institute, Centre for Studies on the Prevention of Alzheimer’s Disease (StoP‐AD), Montréal, QC Canada; ^4^ Lund University, Lund Sweden; ^5^ McGill University, Montreal, QC Canada; ^6^ Otto von Guericke University, Magdeburg Germany

## Abstract

**Background:**

The posterior‐medial network is crucial for episodic memory. However, the medial temporal lobe (MTL) and posteromedial cortex (PMC) regions are vulnerable to aging and early Alzheimer’s disease (AD). Both processes might elicit distinct early functional connectivity (FC) changes which could be detrimental or protective/ compensatory regarding cognition. However, this is not well understood. We hypothesized that resting‐state FC strength between key regions (Figure 1a) would decrease with age and memory decline without AD pathology (A‐T‐) but increase with early AD pathology.

**Method:**

We analysed longitudinal 3‐Tesla resting‐state fMRI data from cognitively unimpaired older adults (OA; PREVENT‐AD cohort). We assessed FC at baseline and after 24 months (FU24) in i) CSF or PET Aß‐ and tau‐negative OA (A‐T‐, N = 96, 63±5years, 70 female, 28 APOE4) and ii) Aß and p‐tau CSF‐characterized OA with available longitudinal p‐tau181/Aß1‐42 ratio (N = 65, 63±5years, 45 female, 22 APOE4). First, we investigated effects of age, APOE genotype and p‐tau181/Aß1‐42 ratio on FC controlling for sex and education. Second, we tested the association between baseline FC or change in FC and change in delayed memory recall in multiple regression analyses.

**Result:**

In A‐T‐ OA, FC decreased mainly between regions within the PMC subnetwork over 24 months (Figure 1b). Higher baseline FCwithin‐PMC was related to increasing memory performance over time (p = 0.047; Figure 2a). Longitudinally, increasing FCMTL‐mPFC was associated with increasing memory in APOE4 non‐carriers and decreasing memory in APOE4 carriers (p = 0.016; Figure 2b). In CSF‐characterized OA, p‐tau181/Aß1‐42 ratio at baseline and FU24 was related to increasing FCMTL‐PMC over time (Figure 3a). Higher baseline FCMTL‐PMC was associated with longitudinally increasing memory in APOE4 non‐carriers and decreasing memory in APOE4 carriers (p = 0.028; Figure 3b).

**Conclusion:**

Our results provide novel longitudinal evidence incorporating age, APOE, Aß and tau indicating specific memory‐related FC changes in cognitively unimpaired OA. APOE moderated the effects of FC strength on change in episodic memory performance. Higher FCMTL‐PMC and increasing FCMTL‐mPFC seem to be detrimental in APOE4 carriers but beneficial in APOE4 non‐carriers. Importantly, this effect was observed in A‐T‐ OA, hinting that APOE genotype may affect FC earlier than AD‐related pathology.